# Contribution of critical amino acid residues in the RNA-dependent RNA polymerase to the replication fidelity and viral ribavirin sensitivity of porcine reproductive and respiratory syndrome virus

**DOI:** 10.1186/s13567-025-01517-9

**Published:** 2025-04-19

**Authors:** Xiaoyan Zhang, Ziyin Yang, Zhibang Zhang, Zhisheng Wang, Yipeng Zhao, Taotao Yang, Jinxiang Gong, Kang Feng, Junping He, Qisheng Zheng, Jibo Hou, Pengcheng Li

**Affiliations:** 1 College of Life Sciences and Resource Environment, Laboratory of Animal Pathogenic Microbiology, Yichun University, Yichun, 336000 Jiangxi China; 2 Center of Engineering and Technology for Veterinary Biologicals, National Research, Jiangsu Academy of Agricultural Science, Nanjing, 210014 Jiangsu China; 3 College of Veterinary Medicine, Shanxi Agricultural University, Jinzhong, 030801 Shanxi China

**Keywords:** RdRp, PRRSV, replication fidelity, ribavirin sensitivity

## Abstract

The porcine reproductive and respiratory syndrome virus (PRRSV) has the highest mutation rate of any known RNA virus. The replication fidelity of RNA viruses can be modulated by subtle amino acid changes in the viral RNA-dependent RNA polymerase (RdRp). In our study, two novel amino acid substitutions (V218I and P386S) in the RdRp of PRRSV were identified under the ribavirin selection. A series of mutant viruses with single or double amino acid replacements were generated from high-fidelity PRRSV NJ-Rb and wild-type NJ-a P80 infectious cDNA clones. Subsequently, we evaluated the genetic stability, ribavirin sensitivity, and biological characteristics of the recombinant viruses. Our findings indicated that the mutation frequencies of the recombinant mutants (vI218V, vS386P, and vVP) based on NJ-Rb were significantly increased and that these recombinant viruses exhibited a loss of ribavirin resistance. The high-fidelity virus NJ-Rb was undetectable using a virus titration assay in porcine alveolar macrophages (PAMs). Our in vivo experiments demonstrated that NJ-Rb was nearly incapable of establishing infection and replicating in the lungs. The recombinant mutants vV218I, vP386S, and vIS, based on NJ-a P80, significantly increased replication fidelity and ribavirin resistance. These results indicated that PRRSV RdRp (NSP9) contained fidelity checkpoints. Furthermore, Val218 and Pro386 were identified as critical sites that determined PRRSV’s genetic stability and ribavirin resistance. These findings contribute to understanding how RdRp affects PRRSV’s genetic stability and ribavirin sensitivity and provide a theoretical basis for designing a genetically stable high-fidelity PRRSV vaccine.

## Introduction

Porcine reproductive and respiratory disease (PRRS), also known as mystery swine disease or blue ear disease, has significantly impacted the global swine industry economically since the first reported outbreak in the 1980s [1]. It is estimated that PRRS costs the swine industry at least $600 million annually in the United States alone [2, 3]. Porcine reproductive and respiratory syndrome virus (PRRSV), a single-stranded, positive-sense RNA virus, is the causative pathogen of PRRS and has the highest mutation rate of any reported RNA virus. The calculated rate of PRRSV nucleotide substitution is 4.7–9.8 × 10^˗2^/site/year, while other RNA viruses typically have a mutation rate within the range of 10^˗3^ to 10^˗5^/site/year [4]. Due to the rapid evolution of PRRSV, there is a significant amount of genetic variability among its strains. The virus shows remarkable genetic variation with two geographically distinct genotypes: European (type 1) and North American (type 2). Furthermore, high levels of genetic variability exist among viruses, even within the same genotype [5]. Currently, modified live-attenuated vaccines are used globally to control this disease. However, it is important to note that live vaccines can pose potential safety risks and may contribute to increased genetic mutations in PRRSV [6].

A quasispecies is a collection of genetically related variants linked through mutations. These variants interact cooperatively on a functional level and collectively contribute to the overall characteristics of the population [7]. The quasispecies diversity of RNA viruses is mainly determined by the fidelity of the RNA-dependent RNA polymerase (RdRp) during viral RNA replication [8–10]. High-fidelity variants of these viruses have been obtained by leveraging the general susceptibility of RNA viruses to mutagens such as ribavirin, as seen in the cases of poliovirus (PV) [11], foot-and-mouth disease virus (FMDV) [12], and Chikungunya virus (CHIKV) [13]. Studies of RNA viruses have shown that certain amino acid residues in RdRp act as fidelity checkpoints and that RdRp fidelity can be modulated by changes in these checkpoint residues [9, 14, 15]. Three crucial functional domains in the PV RdRp regulate viral replication fidelity: the Palm N-terminal (G64S, M296I, and H273R) region, Palm motif A, and K359 and F363 in Palm motif D [9, 14]. Similar to the G64S domain in PV, the Palm N-terminal M296I mutant in FMDV RdRp and the Palm motif D K360R mutant in coxsackievirus (CV) RdRp exhibit higher genetic stability [15]. A possible mechanism for this increased genetic stability is closing the RdRp active site, allowing these viruses to fine-tune replication fidelity and quasispecies distribution easily [9].

PRRSV also exists as a quasispecies during natural infection [16]. Additionally, PRRSV ribavirin-resistant mutants have been reported to be genetically more stable than their parental virus [17, 18]. Nonetheless, the mechanisms and genetic determinants responsible for the fidelity of ribavirin-resistant PRRSV remain unclear. Likewise, whether PRRSV RdRp determines the virus’s replication fidelity, ribavirin sensitivity, and the related mechanisms involved remains ambiguous. The RdRp of PRRSV comprises a catalytic subunit known as non-structural protein 9 (NSP9), which may include the fidelity checkpoints [19]. Subsequently, the genetic diversity of PRRSV constitutes a critical obstacle in PRRS control. A comprehensive investigation into the mechanisms that influence the genetic stability of PRRSV is therefore crucial for fully understanding the root causes of PRRS and for establishing a solid theoretical foundation for the development of safer vaccines.

In this study, we aim to identify the checkpoint amino acid residues in the RdRp of PRRSV that play a crucial role in the fidelity replication of PRRSV and its ribavirin sensitivity. Ribavirin-resistant PRRSV strains were obtained by consecutive passage in MARC-145 cells under the selection of ribavirin. After evaluating genetic stability, it was confirmed that these strains exhibit high fidelity. To verify whether the RdRp of PRRSV is involved in conferring ribavirin resistance and replication fidelity, recombinant infectious clones containing the introduced mutation(s) of PRRSV were constructed. Critical sites that affected the genetic stability and ribavirin sensitivity in PRRSV RdRp were then determined through genetic stability evaluation. Combined with PRRSV RdRp structure prediction, our findings revealed a potential mechanism by which RdRp modulates the genetic stability of PRRSV.

## Materials and methods

### Cells, viruses, reagents, and antibodies

MARC-145 cells were cultured in Dulbecco’s modified Eagle’s medium (DMEM, Gibco, USA) supplemented with 10% (v/v) heat-inactivated foetal bovine serum (FBS) (Gibco, USA) at 37 °C in a humidified 5% CO_2_ incubator. PAMs were collected from six-week-old PRRSV-free pigs via bronchoalveolar lavage [18] and maintained in RPMI 1640 medium supplemented with heat-inactivated 10% FBS at 37 °C in a humidified 5% CO_2_ incubator. Virus-infected cells were grown in DMEM or RPMI 1640 containing 2% FBS. CD163-transfected immortalised PAM cells (3D4/21) were generously provided by Prof. Chunhe Guo (South China Agricultural University).

The PRRSV NJ-a strain [20] was kindly provided by the National Research Center of Engineering and Technology for Veterinary Biologicals (Jiangsu Academy of Agricultural Science, Nanjing, China) and propagated in MARC-145 cells. The PRRSV NJ-a P80 strain (GenBank accession number PP105538.1) was acquired through serial passaging in our lab. NJ-a P80, an attenuated strain, was developed by the serial passage of highly pathogenic PRRSV NJ-a in MARC-145 cells.

The following chemicals were obtained from Sigma-Aldrich: ribavirin (R9644-50MG), guanidine hydrochloride (G3272-500G), and agarose with a low gelling temperature (A9045-10G). PrimeSTAR^®^ Max DNA Polymerase, PrimeScript™ II 1st Strand cDNA Synthesis Kit, RNAiso Plus, MiniBEST Viral RNA/DNA Extraction Kit, T4 DNA Ligase, and DNA markers were purchased from Takara Biomedical Technology Co., Ltd. (Japan). Restriction enzymes were purchased from New England Biolabs (Beijing, China), and FuGENE^®^ HD Transfection Reagent was purchased from Promega (Beijing, China) Biotech Co., Ltd (Promega). Maxima H Minus Double-Stranded cDNA Synthesis Kit was acquired from Thermo Fisher Scientific Inc (USA). The anti-PRRSV nucleocapsid (N) protein monoclonal antibody was prepared in our laboratory. The goat anti-mouse Alexa Fluor 488 (molecular probes) secondary antibody used for indirect immunofluorescence was purchased from Abmart Shanghai Co., Ltd. (Abmart, China).

### Rescue ribavirin-resistant mutant virus

Briefly, the PRRSV NJ-a P80 strain was serially passaged in cell culture in the presence of 200 μM (non-toxic mutagen concentration) ribavirin to study the emergence of ribavirin-resistant PRRSV mutants. Confluent monolayers of MARC-145 cells were prepared in 6-well plates and pre-treated before infection with DMEM growth medium containing ribavirin for 2 h at 37 °C. After pre-treatment incubation, cells were inoculated with PRRSV NJ-a P80 at a multiplicity of infection (MOI) of 0.1. After 1 h of infection, the inoculum was aspirated and washed twice with phosphate-buffered saline (PBS). The cells were then replenished in a DMEM maintenance medium containing the same concentrations of ribavirin for post-treatment. The infection was allowed to proceed for 48 h, after which the cell culture fluid was collected from each well, centrifuged, and stored at −80 °C until use. The supernatant from each passage became the inoculum for the next passage.

### Isolation and identification of mutagen-resistant variants

To isolate and identify mutagen-resistant variants [21], we performed large population-size passages. Subsequently, we assessed virus titres throughout the passage series before isolating the ribavirin-resistant variants after 30 passages by plaque assay.

A standard plaque assay was performed under an agarose (1% final wt/vol) overlay in 6-well plates to isolate the identified mutant. Based on the stock titres, serial dilutions of the virus to dilutions that will produce between 10 and 50 well-separated plaques were prepared. When plaques were clearly visible (typically two to four days post-infection (dpi)), the positions of the plaques on the plates were marked. A p200 pipette with a filter tip was carefully used to penetrate the agarose overlay to avoid dislodging or shifting its position. The agarose plug enclosed in the pipette filter tip was then transferred to an Eppendorf containing 200 μL of medium and vortex. All samples were frozen and thawed thrice. Three rounds of plaque purifications were conducted to isolate the ribavirin-selected P30 virus of the PRRSV strain NJ-a P80.

Next, using the plaque-purified sample mentioned earlier, a larger stock volume for reverse transcription polymerase chain reaction (RT-PCR) was prepared to infect a T25 flask of MARC-145 cells. NSP2 and ORF5, which are known to be the most variable regions in the PRRSV genome [17], were then submitted for sequencing. PCR amplification and sequencing primers are shown in Table 1. The sequences were analysed using the SnapGene software 6.0.2.

**Table 1:** **Sequences of primers used for PCR amplification of the ORF5, NSP2 and NSP9 regions in the PRRSV genome**

Sequenced region	Primer name	Sequences (5*'*-3*'*)	Sequenced length (bp)
ORF5	GP5-F	atgttggggaagtgcttgacc	603
	GP5-R	ctagggacgaccccatagttccgct	
NSP2	NSP2-F	gccggaaaaagagcaaggaaaacac	2850
	NSP2-R	gcccagtaacctgccaagaatgg	
NSP9	NSP9-F	tttaaactgctagccgccagcg	1929
	NSP9-R	ctcatgattggacctgagtttttcccac	

The isolated clones and a wild-type control virus were prepared under similar conditions to confirm the ribavirin resistance conferred by the identified mutation. The previous experiments were repeated using 200 μM ribavirin. Before infection, confluent monolayers of MARC-145 cells were prepared in 24-well plates and pre-treated with DMEM growth medium containing ribavirin for 2 h at 37 °C. Following the pre-treatment incubation, cells were inoculated with PRRSV clones at a MOI of 1. After 1 h of infection, the aspirated inoculum was washed twice with PBS, and cells were replenished in a DMEM maintenance medium containing the same concentrations of ribavirin for post-treatment. The infection was then allowed to proceed for 48 h. Viruses were then released from cells by the freeze–thaw method, and the viral titre in the lysate supernatant was determined by TCID_50_ assay.

### Guanidine-resistance assay

To isolate the mutants with higher replication fidelity [22, 23], we assessed the guanidine sensitivities of plaque-purified ribavirin-resistant populations, which were identified by their growth (0.5 mM) with or without guanidine for 48 h. Briefly, the plaque-purified populations were selected using plaque assays described above and propagated in MARC-145 cells twice to titres of more than 10^7.0^TCID_50_/mL. A total of 2 × 10^5^ MARC-145 cells per well in 24-well plates were either untreated or treated with 0.5 mM guanidine for 2 h at 37 °C in an incubator. The cells were then infected at an MOI of 0.5 and incubated for another 48 h. Viruses were released from the cells using the freeze–thaw method, and the viral titre in the lysate supernatant was determined by TCID_50_ assay.

### Measure mutation frequencies of a potential high-fidelity ribavirin-resistant strain

The ribavirin-resistant mutant strain (high-fidelity strain PRRSV NJ-Rb) and PRRSV NJ-a P110 (PRRSV NJ-Rc) were passaged an additional 10 times. Both PRRSV NJ-Rb and PRRSV NJ-Rc are plaque-purified monoclonal clones; therefore, plaque purification was performed on the supernatants collected after the 10 passages to isolate 50 plaque clones per viral strain (PRRSV NJ-Rb P10 and PRRSV NJ-Rc P10). A short amplification (one replication cycle) on a minimum number of cells (24-well plate format) was used to obtain sufficient RNA for amplification by RT-PCR. Particularly, each clone and population requiring comparison underwent the same number of replication cycles. Viral RNA was extracted from each virus clone using a commercial kit (MiniBEST Viral RNA/DNA Extraction Kit, Takara, Japan) according to the manufacturer’s instructions. NSP2 and ORF5 were amplified using a PrimeScript™ II 1st Strand cDNA Synthesis Kit (Takara, Japan) and were submitted for sequencing.

### Ultra-deep sequencing

To confirm the above results**,** the quasispecies population variants of PRRSV NJ-Rb P10 and PRRSV NJ-Rc P10 were detected by next-generation sequencing. RNA was extracted from the PRRSV NJ-Rb and PRRSV NJ-Rc viruses, and RT-PCR was conducted to allow for the sequencing of the entire genome for full-length assembly. The two resulting sequences were used as the parental reference. According to the manufacturer’s instructions, the RNA samples were reverse transcribed by random hexamers using the Maxima H Minus Double-Stranded cDNA Synthesis Kit (Thermo Fisher Scientific Inc, USA).

The Illumina sequencing and library construction were performed as previously described [24]. In brief, the NEBNext^®^ MLtra™ DNA Library Prep Kit for Illumina^®^, NEB#7370 (NEB, Ipswich, MA, USA) was used for library construction. After adapter ligation, we performed 10 cycles of PCR amplification for sequencing target enrichment. The libraries were pooled at equal molar ratios, denatured, and diluted to optimal concentration before sequencing. The Illumina Hiseq4000 (Illumina, San Diego, CA, USA) was used for sequencing to generate pair-end 150 bp reads.

### Cell tropism assay

Virus titres were measured in PAMs and MARC-145 cells, with calculations based on the cytopathic effect (CPE), and expressed as TCID_50_/mL. The virus titres of each viral clone were then compared in PAMs and MARC-145 cells to see whether there was a change in cell preference.

In vivo experiments were performed to determine whether NJ-Rb replicates in pigs. Six four-week-old PRRSV-negative pigs were randomly divided into two groups for treatment with each virus (NJ-Rb and NJ-Rc). The pigs were injected intramuscularly with each virus at a 1.0 × 10^5.0^ TCID_50_/mL titre at 2.0 mL/pig and observed daily for clinical signs before feeding, up to 28 days post-challenge (dpc). At the end of the 28-day observation period, all pigs were euthanised for necropsy. An array of tissue samples, including lungs, tonsils, bronchial lymph nodes, and muscles, were collected and stored at −80 °C until laboratory processing. The residual virus loads in different tissues were quantified by a real-time reverse transcription PCR (RT-PCR) using TaqMan chemistry, as previously described [18]. A standard curve previously created from known virus titres was used to calculate the amount of PRRSV in each sample by converting the threshold cycle (*C*_*T*_) values to virus titres (TCID_50_/mL).

### Rescue high-fidelity strain and its mutant viruses

The two amino acid mutations were introduced to verify further the involvement of the amino acid mutations (V218I and P386S) in NSP9 of PRRSV to confer replication fidelity and ribavirin resistance. The amino acid mutations were introduced, individually or in combination, into the backbone of an infectious PRRSV clone, which was constructed based on the PRRSV NJ-Rb strain. The recombinant clones containing the introduced mutation(s) were used to transfect fresh MARC-145 cells using the FuGENE^®^ HD transfection reagent (Promega) according to the manufacturer’s instructions. The rescued reconstituted viruses (P3), vI218V (containing the I218V substitution), vS386P (S386P), vVP (I218V and S386P), and vNJ-Rb were all viable. Viability was evidenced by staining using the anti-PRRSV N protein immunofluorescence assay monoclonal antibody. One-step virus growth kinetics and cell tropism of these strains were then tested. The rescued reconstituted viruses, vV218I (containing the V218I substitution), vP386S (P386S), vIS (V218I and P386S), and vNJ-a P80 were constructed based on the PRRSV NJ-a P80 strain.

The immunofluorescence assay (IFA) was performed as described by Li et al. [25]. In brief, MARC-145 cells were differentiated on glass coverslips, which were placed in 24-well tissue culture plates until each well was 75% confluent. MARC-145 were infected for 24 h with the rescued reconstituted viruses. The cell preparations were fixed for 15 min at room temperature in PBS-4% paraformaldehyde. The fixed cells were then permeabilised with 0.1% (v/v) Triton X-100 in PBS for 5 min at room temperature. After being washed thrice with PBS, the cells were blocked with 1% bovine serum albumin at 37 °C for 30 min. Subsequently, they were inoculated with anti-PRRSV N protein monoclonal antibody (1:500) overnight at 4 °C and washed thrice with PBS. Antibody binding was detected using secondary antibodies conjugated with Alexa Fluor 488 for 1 h in a moist container and kept in the dark at 37 °C. The cells were then stained with DAPI (0.1 μg/mL) for 10 min at room temperature. Finally, the coverslips were mounted on the microslides and air-dried. Images were visualised and captured on a confocal fluorescence microscope (ZEISS 710, Germany) or a fluorescence microscope (Nikon Eclipse Ci, Japan).

### Evaluation of the RdRp of PRRSV mutants for binding to CD163

The mutations in PRRSV RdRp were analysed to see if they affected their binding to CD163. CD163-transfected immortalised PAM cells (3D4/21) were differentiated on glass coverslips, which were placed in 24-well tissue culture plates until each well reached 75% confluent. The cells were then infected with the mutated viruses (vI218V, vS386P vVP, vNJ-Rb, NJ-Rb, and NJ-Rc) for 24 h. Viruses were IFA-stained with anti-PRRSV N protein monoclonal antibody.

### Assessment of genetic stability of the recombinant viruses during passages in MARC-145 cells

To assess the genetic stability of the recombinant viruses (vI218V, vS386P vVP, and vNJ-Rb) derived from sequential passages of PRRSV NJ-Rb, they were passaged an additional 30 times, alongside PRRSV NJ-Rb. Plaque purification was conducted on the supernatants collected after 30 passages to isolate 50 plaque clones per viral strain. RT-PCR was performed to amplify and purify the PCR products (NSP2 and ORF5), with subsequent sequencing to conduct a mutation analysis. Genetic stability assays of the recombinant viruses (vV218I, vP386S vIS, and vNJ-a P80) were performed as described above.

### Assessment of ribavirin resistance of the recombinant viruses

The recombinant viruses P2 were inoculated in the presence of ribavirin, as described above, in MARC-145 cells, along with NJ-Rb or NJ-a P80 as the control. The viruses released from the cells were collected after 48 h by the freeze–thaw method, and the viral titre in the lysate supernatant was determined by TCID_50_ assay.

### Structural prediction and analysis of PRRSV RdRp

The three-dimensional structure of PRRSV RdRp was simulated by the AlphaFold Colab (Version 2.1.0) online prediction tool [26]. The predicted structure was then compared with the DALI server. The structural results of the simulation and comparison were observed using PyMOL (Version 2.3.2), and the key amino acid sites were analysed.

### Data analysis

GraphPad Prism 5.0.2 (GraphPad Software, Inc., San Diego, CA, USA) was used to create the graphs. Statistical analysis was performed using SPSS Advanced Statistics 16.0 software (SPSS, Inc., Chicago, IL, USA). All values represent the mean of at least three independent experiments. Results were expressed as means ± SEM. ANOVA and unpaired Student’s *t*-test were employed to determine statistical differences among multiple groups. Nucleotide sequences were aligned and analysed using Lasergene^®^ MegAlign software (DNASTAR Inc., Madison, WI, USA).

## Results

### Emergence of ribavirin-resistant variants after successive passage of PRRSV in MARC-145 cells under ribavirin selection pressure

The PRRSV NJ-a P80 was serially passaged in MARC-145 cells in the presence of ribavirin at a concentration of 200 μM. The viral titres exhibited a significant decrease, followed by a gradual recovery. Our results showed that the concentration of 200 μM ribavirin could suppress virus replication to low levels based on a virus titration assay at passages (P) 6–10. Notably, the titres decreased by more than 100 000-fold compared with the control virus without ribavirin. Starting from P10, the virus titre gradually increased and stabilised from P18 to P30. However, the viral titres remained ~ 2-log lower than the control virus without ribavirin.

### Mutation distribution summary for the ribavirin-resistant mutants

Fifty virus clones were plaque-purified from each supernatant of the ribavirin-resistant mutants for each virus strain (PRRSV NJ P110) with or without ribavirin. The hypervariable regions (ORF5 and NSP2) of the 50 virus clones were subsequently amplified for sequencing. Interestingly, after the addition of ribavirin, the nucleotide mutation rate in ORF5 decreased from 6.33 to 3.35 (/10^3^ nt), and the amino acid mutation rate also reduced from 7.76 to 5.07 (/10^3^ aa) (Table 2). Furthermore, ribavirin was shown to have a mutagenic effect on NSP2, leading to a significant increase in mutations in both the nucleotides and amino acids of NSP2. Specifically, the amino acid mutation rate increased by ~2.5 times, from 4.32 to 10.57 (/10^3^ aa). However, the total numbers of the ORF5 and NSP2 mutation sites significantly decreased. In the PRRSV NJ P110 virus without ribavirin, ORF5 contained 21 nucleotides and six amino acid mutation sites (Table 2).

**Table 2:** **Mutation frequencies in the ORF5 and NSP2 regions of the potential ribavirin-resistant mutant viruses**

Sequenced region		(−Rb) P30^a^	(+Rb) P30
ORF5	Total no. of clones sequenced	50	50
	Total no. of nucleotides sequenced (603 nt per clone)	30,150	30,150
	Total no. of mutations	191 (21 loci)	101 (3 loci)
	Mutation rate/10^3^ nt	6.33	3.35**
	Total no. of amino acids sequenced (201 aa per clone)	10,050	10,050
	Total no. of mutations	78 (6 loci)	51 (2 loci)
	Mutation rate/10^3^ aa	7.76	5.07**
NSP2	Total no. of clones sequenced	50	50
	Total no. of nucleotides sequenced (2850 nt per clone)	142,500	142,500
	Total no. of mutations	343 (72 loci)	659 (23 loci)
	Mutation rate/10^3^ nt	2.41	4.62***
	Total no. of amino acids sequenced (950 aa per clone)	47,500	47,500
	Total no. of mutations	205 (44 loci)	502 (15 loci)
	Mutation rate/10^3^ aa	4.32	10.57***

^a^The PRRSV strain NJ-a P80 viral clone was serially passaged for 30 passages (P30) with (+Rb) or without (−Rb) 200 μM ribavirin. nt: nucleotide, aa: amino acid.

Asterisks represent significant differences at **P < 0.01, ***P < 0.001.

In contrast, PRRSV NJ P110, treated with ribavirin, contained only three nucleotide mutation sites and two amino acid mutation sites in ORF5 (Table 2). In the PRRSV NJ P110 virus without ribavirin, NSP2 exhibited 72 nucleotide and 44 amino acid mutation sites. Meanwhile, PRRSV NJ P110, treated with ribavirin, contained only 23 nucleotide mutation sites and 15 amino acid mutation sites in NSP2 (Table 2). The distribution of mutations in the PRRSV RdRp (NSP9) is presented in Table 3. In PRRSV NJ P110, which was not treated with ribavirin, there were 10 nucleotide mutation sites and two amino acid mutation sites in the RdRp. Only two of the 20 clones were mutated, which were single-point mutated, each containing one mutation site. The results showed that RdRp is highly conserved. In contrast, the PRRSV NJ P110 treated with ribavirin had five nucleotide mutation sites and two amino acid mutation sites in RdRp. All cloned viruses exhibited amino acid mutations at two specific sites, V218I and P386S.

**Table 3:** **Distribution of mutations in the RdRp (NSP9) regions**

Sequenced region	Viruses	aa# in amino acid	Mutation	# clones^a^ with n mutations	loci
NSP9 (nucleotides)	(−Rb) P30	135	T–C	1	10
		477	A–G	6	
		801	C–T	17	
		1110	C–T	1	
		1125	C–T	1	
		1206	C–T	1	
		1472	A–G	1	
		1530	C–T	1	
		1629	A–G	1	
		1719	C–T	1	
		258	T–C	1	5
	(+Rb) P30	462	G–A	20	
		652	G–A	20	
		1156	C–T	20	
		1533	T–C	20	
NSP9 (amino acids)	(−Rb) P30	160	Q–H	1	2
		491	D–G	1	
	(+Rb) P30	218	V–I	20	2
		386	P–S	20	

^a^Total of 20 plaque clones per viral strain were sequenced.

### PRRSV NJ-Rb has the potential for high fidelity

**Figure 1:**
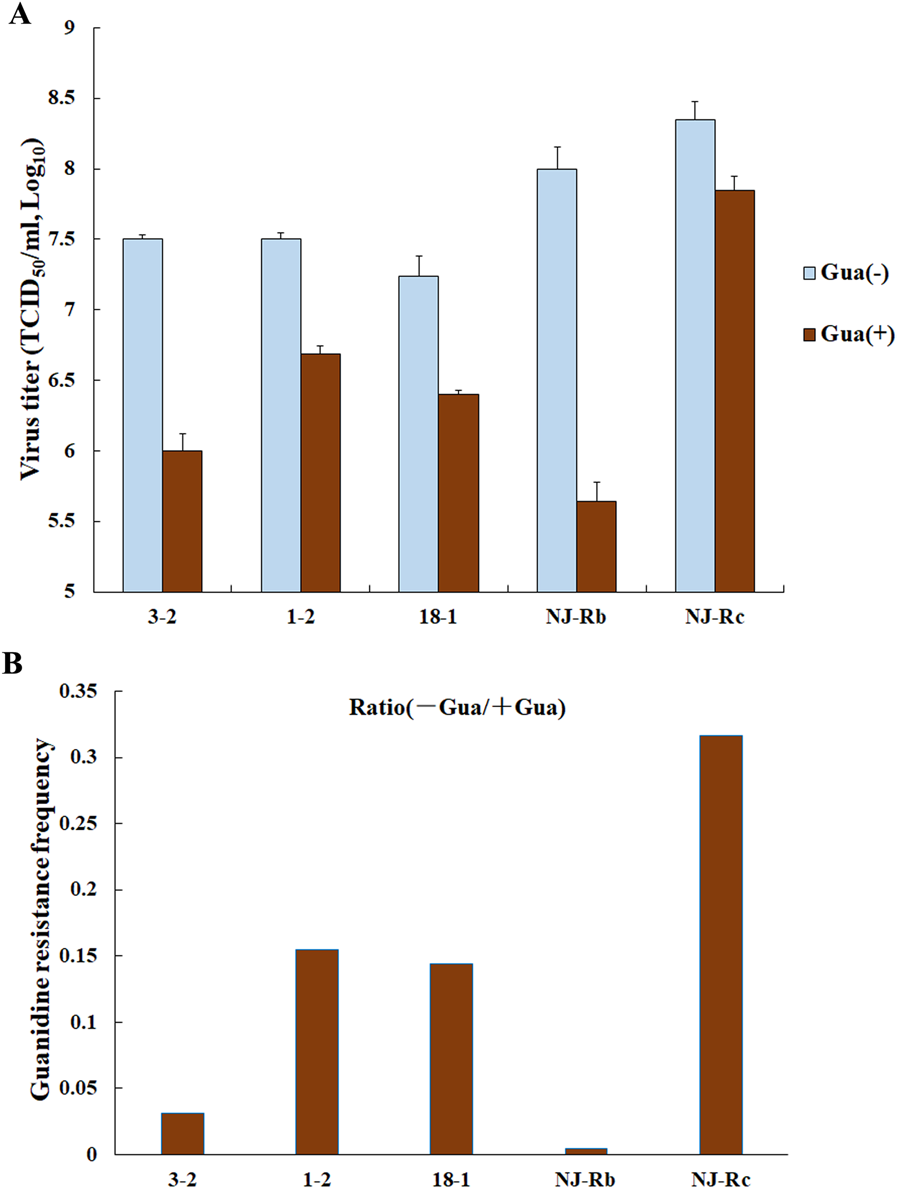
**Comparative fidelity of plaque-purified viral populations and wild-type virus (NJ-Rc).** Samples of the plaque-purified populations (3–2, 1–2, 18–1 and NJ-Rb) and NJ-Rc were cultured for 48 h in MARC-145 cells (MOI, 0.5) in the presence or absence of 0.5 mM guanidine (Gua). We compared the selected clones with or without 0.5 mM guanidine and found one clone, designated NJ-Rb, had the highest sensitivity to guanidine and the largest decrease in viral titre from 10^8 ± 0.15^ TCID_50_/mL to 10^5.64 ± 0.13^ TCID_50_/mL. **A** The resulting viral titres were determined using a TCID_50_ assay on MARC-145 cells. The experiment was performed in triplicate, and each error bar indicates one standard error of the mean (1 SEM). **B** The guanidine resistance ratios of the plaque-purified populations, NJ-Rb and NJ-Rc, were calculated as the viral titre in the presence of guanidine divided by the titre in the absence of guanidine.

We conducted a guanidine-resistance assay to test the hypothesis that ribavirin-resistant mutants exhibit increased fidelity in RNA synthesis compared to the wild-type virus [22, 23]. An estimation of error frequency was obtained by measuring the number of resistant viruses in a population. We compared the selected clones, with or without 0.5 mM guanidine, and found that one of the clones, designated NJ-Rb, had the highest sensitivity to guanidine, showing the largest decrease in viral titre from 10^8 ± 0.15^ TCID_50_/mL to 10^5.64 ± 0.13^ TCID_50_/mL (Figure 1A). Furthermore, NJ-Rb also had the lowest apparent error frequency of guanidine resistance, which was 72.44-fold lower than that of the wild-type strain NJ-Rc (plaque-purified virus) (Figure 1B). These results suggested that NJ-Rb is a potential high-fidelity strain.

### PRRSV NJ-Rb demonstrates promising stability in MARC-145 cells

**Figure 2:**
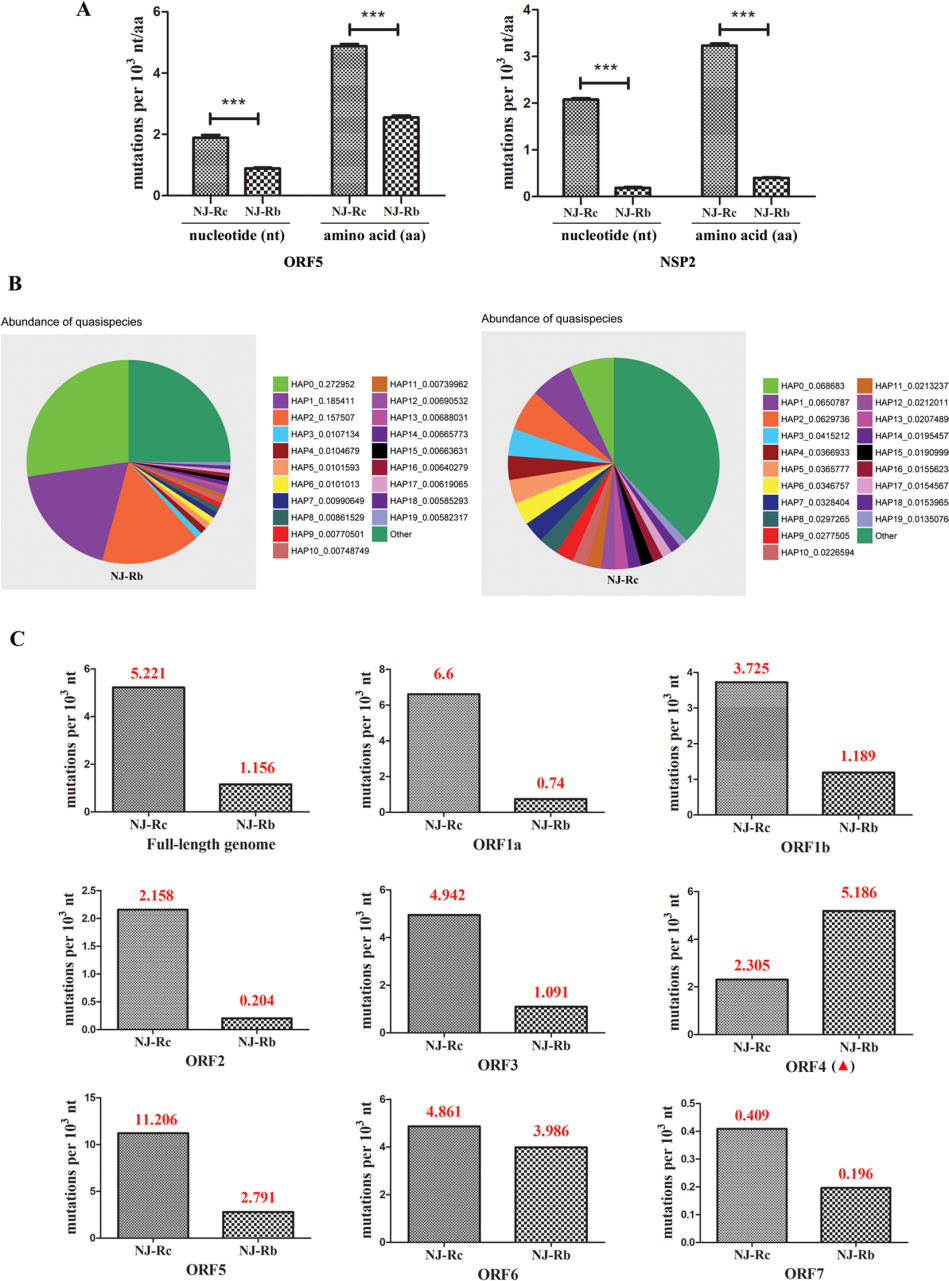
**Comparative mutation frequencies of PRRSV NJ-Rb and NJ-Rc (wild-type).** **A** Mutation frequencies in the ORF5 and NSP2 regions of plaque-cloned, ribavirin-resistant mutant viruses (PRRSV NJ-Rb) after 10 passages in MARC-145 cells. Statistical significance was evaluated by determining the *P*-values. ***P < 0.001. **B** Abundance of quasispecies of NJ-Rb and NJ-Rc. The GeneID indicates the quasispecies ID, along with its sequence number (e.g., HAP0 ~ 19) and abundance information (e.g., 0.272952). The top 20 most abundant quasispecies were counted, and the rest were categorised as “Other”. The cumulative abundance of NJ-Rb quasispecies was more than 99.99%, and that of NJ quasispecies was more than 99.63%. (C) Full-length genome and ORF mutation frequency analysis. The red numbers represent the value. Except for ORF4 (red arrow), the mutation frequencies of the segmental genes in NJ-Rb, ORF1a, ORF1b, ORF2, ORF3, ORF5, ORF6 and ORF7 were significantly decreased.

PRRSV NJ-Rb and NJ-Rc (wild-type) were serially passaged 10 times in MARC-145 cells without ribavirin. After 10 passages, 50 virus clones were isolated from the supernatant of each strain through plaque purification. The ORF5 and NSP2 regions of these clones were amplified for sequencing. The results showed that NJ-Rb exhibited lower mutation frequencies than NJ-Rc after 10 sequential passages in MARC-145 cells (Figure 2A). A total of 56 nucleotides (7 loci) and 49 amino acids (4 loci) substitutions were identified in the ORF5 region of NJ-Rc. Conversely, NJ-Rb had 28 nucleotides (4 loci) and 26 amino acids (2 loci) substitutions in the same region. The mutation frequencies of NJ-Rb in NSP2 were significantly lower than those of NJ-Rc. Specifically, NJ-Rc exhibited 294 nucleotide substitutions at 49 loci and 153 amino acid substitutions at 23 loci, while NJ-Rb showed only 29 nucleotide substitutions at 18 loci and 19 amino acid substitutions at 10 loci. Compared to NJ-Rc, the ORF5 and NSP2 regions of NJ-Rb exhibited a reduction of approximately 2- and 10.3-fold in the number of nucleotide substitutions, as well as a decrease of approximately 1.8- and eightfold in the number of amino acid substitutions (Figure 2A).

PRRSV NJ-Rb and NJ-Rc underwent next-generation sequencing following 10 consecutive passages in MARC-145 cells without ribavirin. Their quasispecies diversities were compared to confirm the results of the above clone sequencing. A total of 140 quasispecies variants of NJ-Rb and 170 quasispecies variants of NJ-Rc were analysed. The data revealed that the top three dominant quasispecies variants of NJ-Rb accounted for over 60%, while those of NJ-Rc constituted merely 19.67% (Figure 2B). With the exception of ORF4, the mutation frequencies of the segmental genes in NJ-Rb (ORF1a, ORF1b, ORF2, ORF3, ORF5, ORF6, and ORF7) were significantly lower. The total mutation frequency of the complete NJ-Rb genome was 4.52-fold lower than that of NJ-Rc (Figure 2C).

### PRRSV NJ-Rb loses tissue tropism in porcine alveolar macrophages

The growth characteristics of the selected ribavirin-resistant clones (2–2, 38–1, 36–2, 25–2, 23–4, and NJ-Rb) were evaluated in porcine alveolar macrophages (PAMs) and MARC-145 cells to determine their replication capabilities in both cell types. As shown in Table 4, the NJ-a virus replicated to similar levels in PAMs and MARC-145 cells, with titres of 10^7.15^ TCID_50_/mL and 10^7.25^ TCID_50_/mL, respectively. The replication of NJ-Rc (wild-type) was significantly lower than that of NJ P80 (parental virus) in PAMs, with virus titres decreasing from 10^4.25^ TCID_50_/mL to 10^2.5^ TCID_50_/mL. However, NJ-Rc could replicate efficiently in MARC-145 cells, with higher titres of 10^7.75^ TCID_50_/mL. Furthermore, although the different resistant clones could replicate efficiently in MARC-145 cells, their replication efficiency in PAMs was significantly reduced. The virus titre of NJ-Rb was undetectable in PAMs (Table 4), and it did not produce typical cytopathic effects (CPEs). These results revealed that NJ-Rb did not replicate in PAMs, and its tissue tropism was significantly altered.

**Table 4:** **In vitro growth characteristics of ribavirin-resistant mutant viruses in PAM and MARC-145 cells**

Clones^b^	Mean titre in PAMs (TCID_50_/mL, Log_10_)	Mean titre in Marc145 (TCID_50_/mL, Log_10_)	PM Difference^a^
2–2	1.6	6.75	−5.15
38–1	1.0	6.75	−5.75
36–2	1.25	6.6	−5.35
25–2	1.0	7.4	−6.4
23–4	1.6	6.64	−5.04
NJ-Rb	0	7.5	−7.5
NJ-Rc	2.5	7.75	−5.25
NJ P80 NJ-a	4.25 7.15	7.12 7.25	−2.87 −0.1

^a^PM difference, difference in virus titres between results for PAMs and MARC-145 cells as determined by subtracting the geometric mean titre (log_10_) of the virus in MARC-145 cells from the geometric mean titre (log_10_) of the virus in PAMs. This value was used to define the cell preference of the viruses.

^b^The results of partial clones are presented here.

### Rescue and biological characterisation of the reconstituted viruses

**Figure 3:**
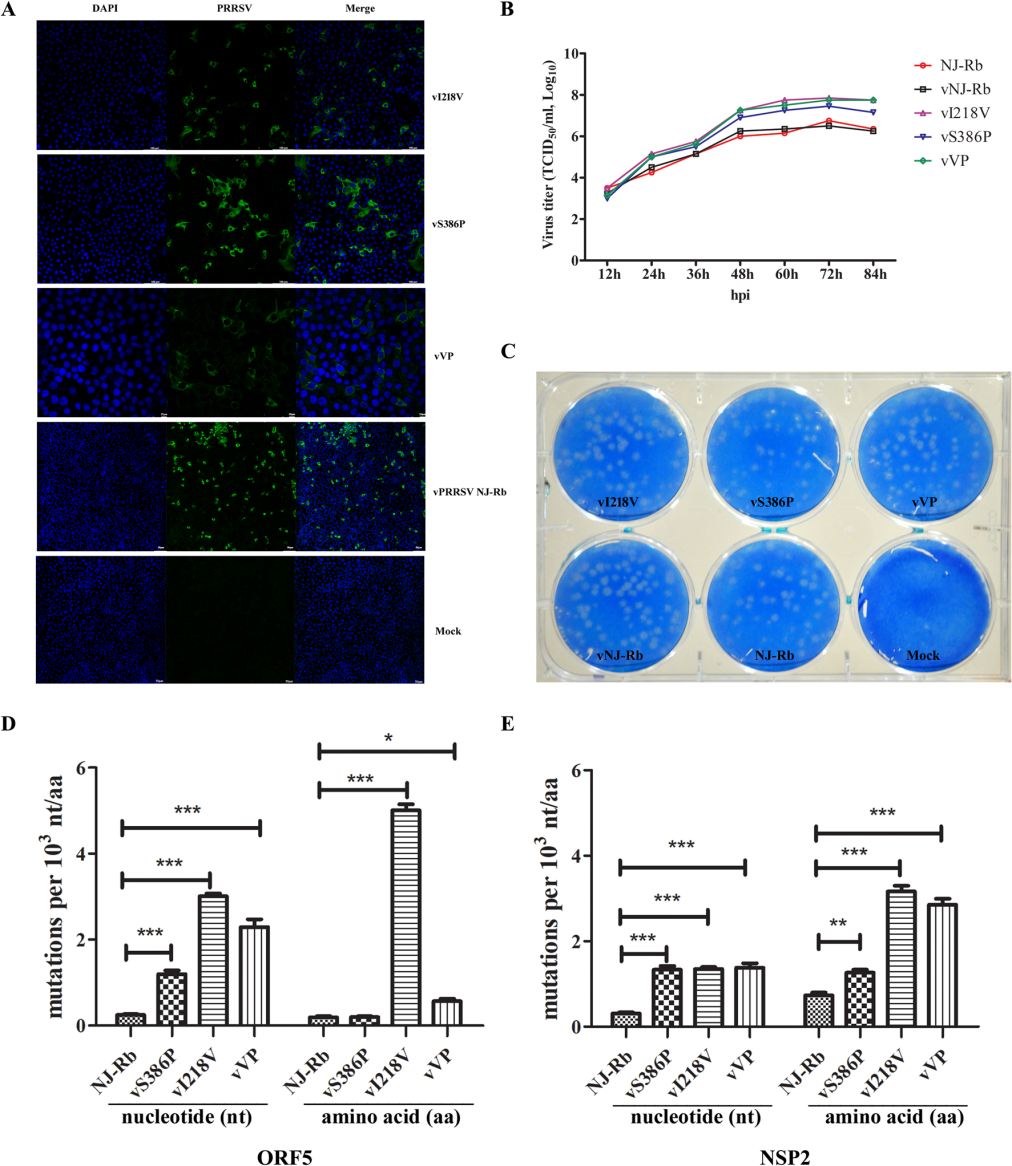
**Rescue, biological characterisation and genetic stability of the reconstituted viruses.** **A** Representative confocal images showing reconstituted viruses infecting MARC-145 cells. Forty-eight h post-transfection of MARC-145 cells with recombinant full-length virus clones, the cell monolayers were immunostained by an immunofluorescence assay. PRRSV was labelled with the N protein primary antibody (green), and nuclei were stained with DAPI (blue). **B** Multiple-step growth curves of viruses. The P2 reconstituted viruses vI218V, vS386P, vVP and vNJ-Rb, and the parental virus NJ-Rb, were used to infect fresh MARC-145 cells at an MOI of 0.1. The culture supernatants were collected and titrated at the indicated time points post-infection (pi). **C** Plaque morphology of viruses. At 4 dpi with the P2 reconstituted viruses (vI218V, vS386P, vVP, vNJ-Rb) and NJ-Rb, the MARC-145 cells were fixed, and plaques were visualised by crystal violet staining. Graphical representation of the mutation ORF5 (**D**) and NSP2 (**E**) frequencies of the reconstituted viruses. Statistical significance was evaluated by determining the *P*-values. *P < 0.05; **P < 0.01, ***P < 0.001.

To further investigate whether the amino acid mutations (V218I and P386S) in the RdRp of PRRSV contribute toward replication fidelity and ribavirin resistance, we introduced the two amino acid mutations individually or in combination into the backbone of an infectious PRRSV clone constructed from the PRRSV strain NJ-Rb. The rescued reconstituted viruses, vI218V (I218V), vS386P (S386P), vVP (I218V and S386P), and vNJ-Rb were all viable, as evidenced by an immunofluorescence assay using an anti-PRRSV N protein monoclonal antibody for staining (Figure 3A). Additionally, passage 3 of the reconstituted viruses and passage 3 of the parental NJ-Rb virus were analysed for their virological characteristics. The reconstituted viruses, vI218V, vS386P, and vVP, displayed lower viral peak titres than the parental virus vNJ-Rb (Figure 3B). Additionally, the titres were not significantly different between vNJ-Rb and NJ-Rb (Figure 3B). Notably, the size and morphology of the plaques produced by all recombinant viruses did not exhibit any significant differences from those produced by the parental virus NJ-Rb (Figure 3C).

### Amino acid residues Val218 and Pro386 in RdRp are involved in regulating the genetic stability of PRRSV NJ-Rb

Sequencing of the clones revealed that the vI218V, vS386P, and vVP viruses generated more mutations than the parental virus NJ-Rb and exhibited a significantly higher mutation frequency (*P* < 0.001) in the ORF5 gene (Figure 3D). The ORF5 amino acid mutation frequency of vI218V was significantly increased by 25-fold, while the mutation frequency of the vVP virus was also significantly increased (*P* < 0.05). In contrast, there was no significant change in the vS386P virus (Figure 3D). Compared to vNJ-Rb, the mutation frequency of the NSP2 gene in the three mutants was significantly increased (*P* < 0.001). Meanwhile, the mutation frequency of the NSP2 amino acid in the vS386P virus was significantly higher (*P* < 0.01). The NSP2 amino acid mutation frequency of vI218V and vVP viruses showed an even more significantly increased ~ fourfold (*P* < 0.001) when compared to the parental virus NJ-Rb (Figure 3E). These results demonstrated that both V218 and P386 are critical amino acid sites affecting the genetic stability of PRRSV, especially V218.

### The amino acid substitutions in RdRp have no impact on PRRSV fitness

**Figure 4:**
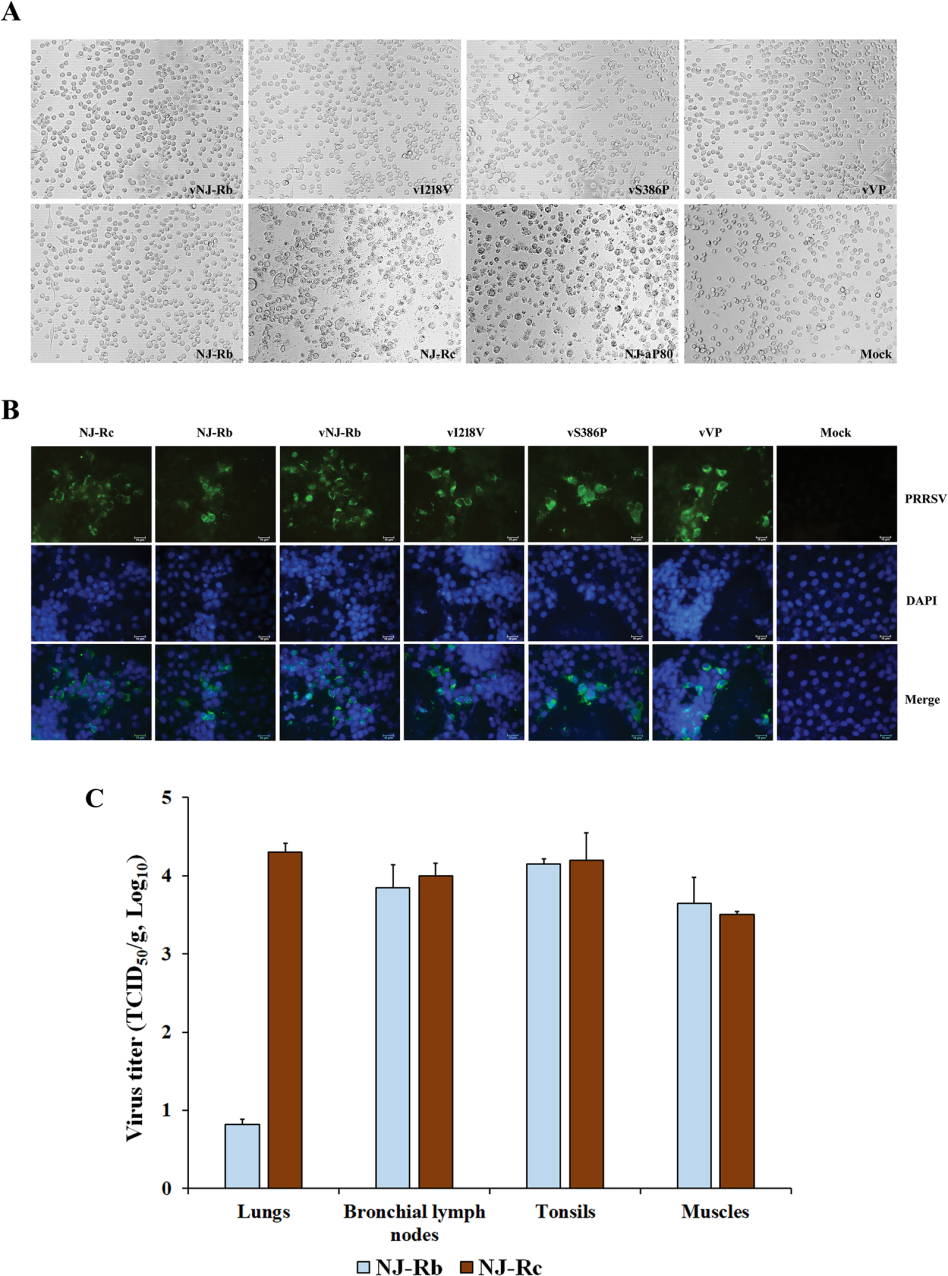
**Observation of the reconstituted viruses and NJ-Rb in viral tissue tropism**. **A** Growth characteristics of the reconstituted viruses (P1), NJ-Rb, NJ-Rc and NJ-a P80 in PAM cells, as observed by microscopy (100 ×). A viral CPE was observed in MARC-145 cells following NJ-Rc and NJ-a P80 infection. **B** Representative fluorescent images showing reconstituted viruses infecting CD163-transfected immortalised PAM cells (3D4/21). The viruses (vI218V, vS386P vVP, vNJ-Rb, NJ-Rb and NJ-Rc) were all viable as evidenced by IFA. PRRSV was labelled with the N protein primary antibody (green), and nuclei were stained with DAPI (blue). **C** Detection of residual virus loads in different tissues in groups challenged with each virus (NJ-Rb and NJ-Rc) via qRT-PCR (TaqMan chemistry). The residual virus loads were measured, and virus titres in different tissues were calculated and expressed as virus titres in TCID_50_/g equivalents based on the standard curve of the cycle threshold (*C*_*T*_) number plotted against the known virus titre of NJ-a p80. Asterisks indicate significant differences in residual virus loads in tissues compared to those of the NJ-Rc group (***P < 0.001).

Similar to the parental virus NJ-Rb, the recombinant viruses vI218V, vS386P, vVP, and vNJ-Rb did not induce a CPE in PAMs and were unable to replicate (Figure 4A). In addition, we found that the mutants (vI218V, vS386P, and vVP) could grow in CD163-transfected immortalised PAM cells (3D4/21) (Figure 4B). These findings indicated that the amino acid substitutions in RdRp did not affect PRRSV fitness in vitro. The in vivo experiments measured residual virus loads in various tissues (lungs, bronchial lymph nodes, tonsils, and muscles) using qRT-PCR, as summarised in Figure 4C. The results indicated that NJ-Rb proliferated effectively in the tonsils, bronchial lymph nodes, and muscles, showing no significant differences compared to NJ-Rc. However, NJ-Rb-challenged pigs had virus titres of 10^0.82 ± 0.07^ TCID_50_/g in the lungs, which were significantly lower (*P* < 0.001) than the virus titres measured in NJ-Rc-challenged pigs (10^4.3 ± 0.12^ TCID_50_/g) (Figure 4C). These results were consistent with the in vitro detection of tissue tropism for the NJ-Rb PAMs.

### vI218V, vS386P, and vVP lost ribavirin resistance

**Figure 5:**
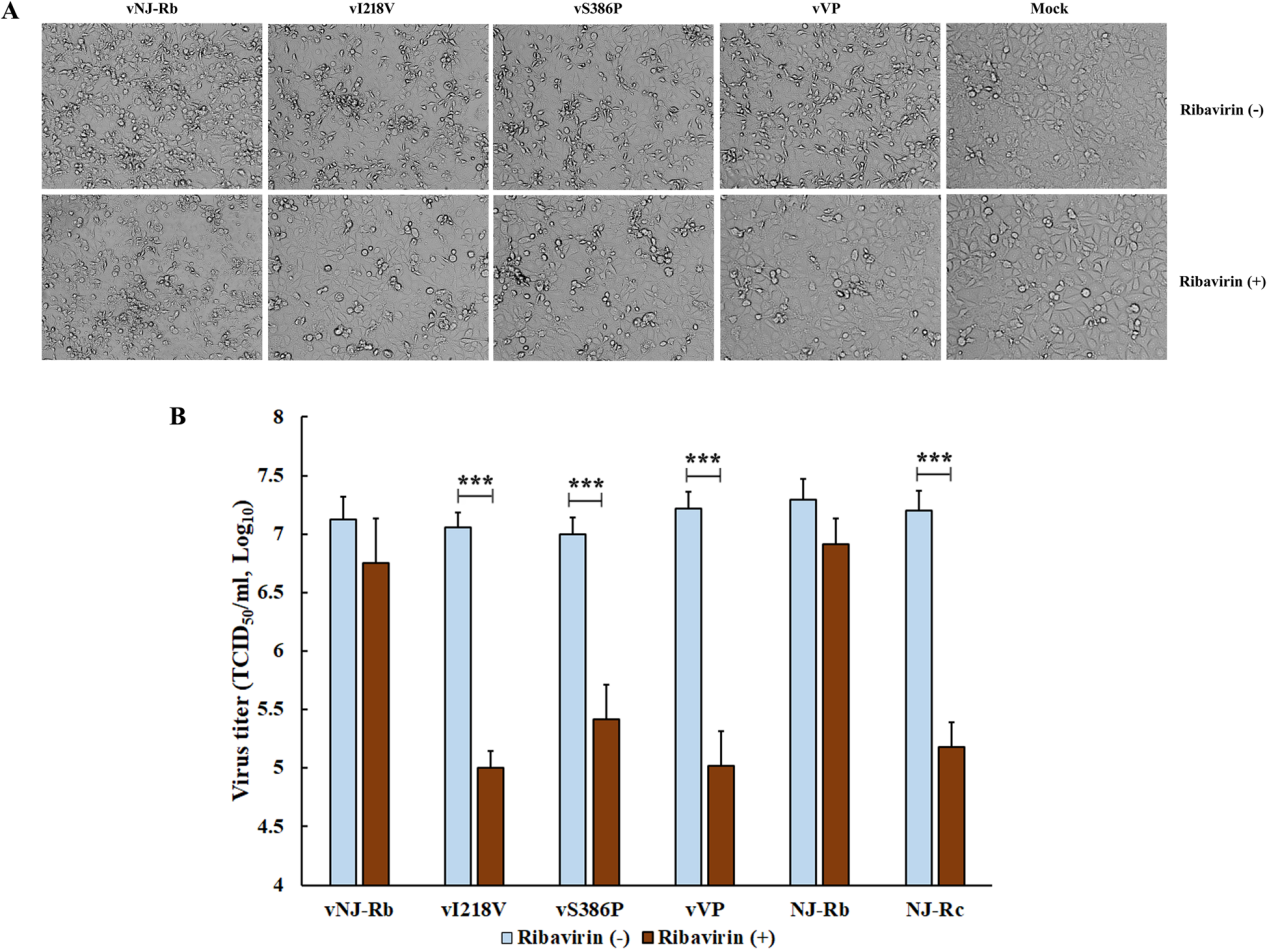
**Ribavirin-resistance profiles of the reconstituted viruses**. **A** Representative images showing reconstituted virus infection in MARC-145 cells in the presence ( +) or absence (−) of 200 μM ribavirin (100 ×). A viral CPE was observed in MARC-145 cells. **B** TCID_50_ assay of the reconstituted viruses with or without ribavirin. Statistical significance was evaluated by determining the *P*-values. ***P < 0.001.

After 48 h of ribavirin treatment, minimal CPEs were observed in MARC-145 cells infected with recombinant viruses vI218V, vS386P, and vVP (Figure 5A). However, infection with the recombinant vNJ-Rb virus showed no significant difference in the extent of CPE in MARC-145 cells, regardless of ribavirin treatment (Figure 5A). The results of virus titre determination were consistent with the visual observation of CPE changes. The reconstituted viruses vI218V, vS386P, and vVP, along with NJ-Rc treated with ribavirin, showed a significant decrease (*P* < 0.001) in viral titre compared to those without ribavirin in MARC-145 cells (Figure 5B). However, no statistically significant difference in viral titre was observed between the recombinant vNJ-Rb virus and its parental strain NJ-Rb when treated with ribavirin in MARC-145 cells (Figure 5B). These results suggest that the two amino acid substitutions in RdRp conferred resistance to ribavirin in PRRSV.

### Confirmation of the recombinant viruses vV218I and vP386S

**Figure 6:**
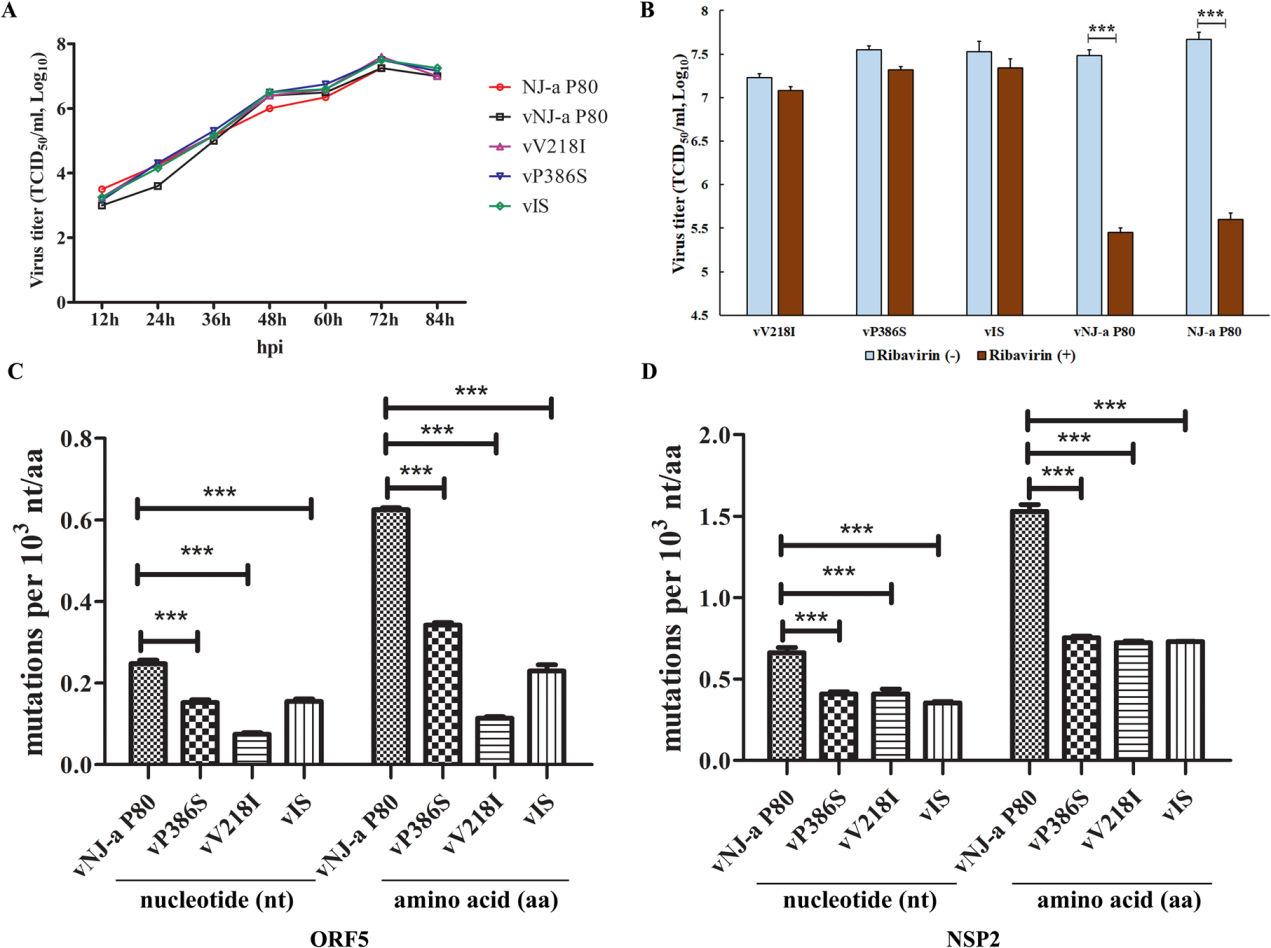
**Construction and evaluation of the recombinant virus V218I and P386S based on NJ-a P80.** **A** Multiple-step growth curves of viruses. The P2 reconstituted viruses vV218I, vP386S, vIS, vNJ-a P80, and the parental virus NJ-a P80 were used to infect fresh MARC-145 cells at an MOI of 0.1. The culture supernatants were collected and titrated at the indicated time points post-infection (pi). **B** TCID_50_ assay of the reconstituted viruses with or without ribavirin. **C** Graphical representation of the mutation ORF5 (**C**) and NSP2 (**D**) frequencies of the reconstituted viruses. Statistical significance was evaluated by determining the *P*-values. ***P < 0.001.

We constructed the recombinant virus with V218I and P386S to support the abovementioned conclusions, using NJ-a P80. The rescued reconstituted viruses, vV218I (V218I), vP386S (P386S), vIS (V218I and P386S), and vNJ-a P80, were all viable as evidenced by IFA (data not shown). The one-step virus growth kinetics results indicated that all recombinant viruses (passage 3) exhibited no significant differences from the parental virus NJ-a P80 (Figure 6A). The reconstituted virus vNJ-a P80, along with NJ-a P80 treated with ribavirin, exhibited a significant decrease (*P* < 0.001) in viral titre compared to those without ribavirin in MARC-145 cells (Figure 6B). However, no statistically significant difference was observed in the recombinant viruses vV218I, vP386S, and vIS treated in MARC-145 cells (Figure 6B), irrespective of treatment with or without ribavirin. These results suggest that the two amino acid substitutions in RdRp conferred resistance to ribavirin in PRRSV and that the mutants (vV218I, vP386S, vIS, and vNJ-a P80) were able to replicate in PAMs (data not shown).

Furthermore, sequencing of the clones revealed that the vV218I, vP386S, and vIS viruses generated fewer mutations than the parental virus NJ-a P80 and presented a significantly lower mutation frequency (*P* < 0.001) in the ORF5 gene and amino acid (Figure 6C). The gene and amino acid mutation frequencies of vV218I were lower (~ onefold) than vP386S (Figure 6C). Compared with vNJ-a P80, the NSP2 gene mutation frequency of the three mutants was significantly decreased (*P* < 0.001) (Figure 6D). The results also showed that both V218 and P386 are critical amino acid sites affecting the genetic stability of PRRSV, especially V218.

### Amino acid residues Val218 and Pro386 in RdRp are conserved in both PRRSV genotypes

**Figure 7:**
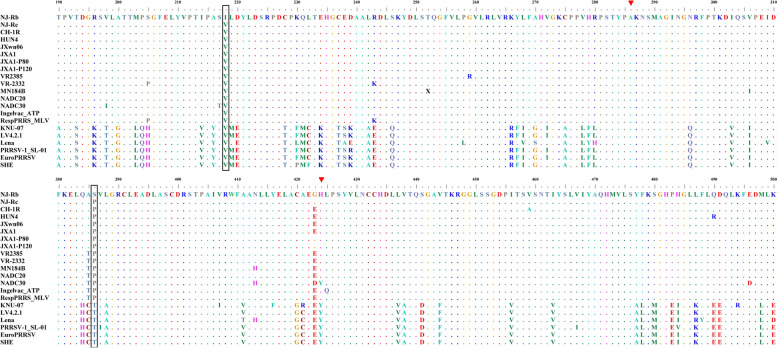
**Sequence analysis of the NSP9 of PRRSVs.** Sequence alignment of selected type 1 and type 2 PRRSV strains. The boxes show amino acids at residues 218 and 386. The red arrows show amino acids at residues 286 and 424. Dots represent identical amino acids with consensus sequences. The alignments were generated using the Cluster W method in the MEGALIGN software (DNASTAR Lasergene).

Thirteen genotype 2 PRRSV strains, from different years of isolation and geographical locations, with different pathogenicity, along with six genotype 1 strains, were selected for RdRp sequence alignment (Figure 7). The sequence alignment data indicated that the residue Val218 was conserved across all selected PRRSV strains. However, the residue identified in PRRSV NJ-Rb was Ile218. Additionally, the residue Pro386 was conserved across all selected PRRSV-2, while the residue identified in PRRSV NJ-Rb was Ser386. At the same position, the residue in all genotype 1 strains was Thr386, although this was also a conserved amino acid site.

### Structural characterisation of RdRp and the location of V218I and P386S in the structural model

**Figure 8:**
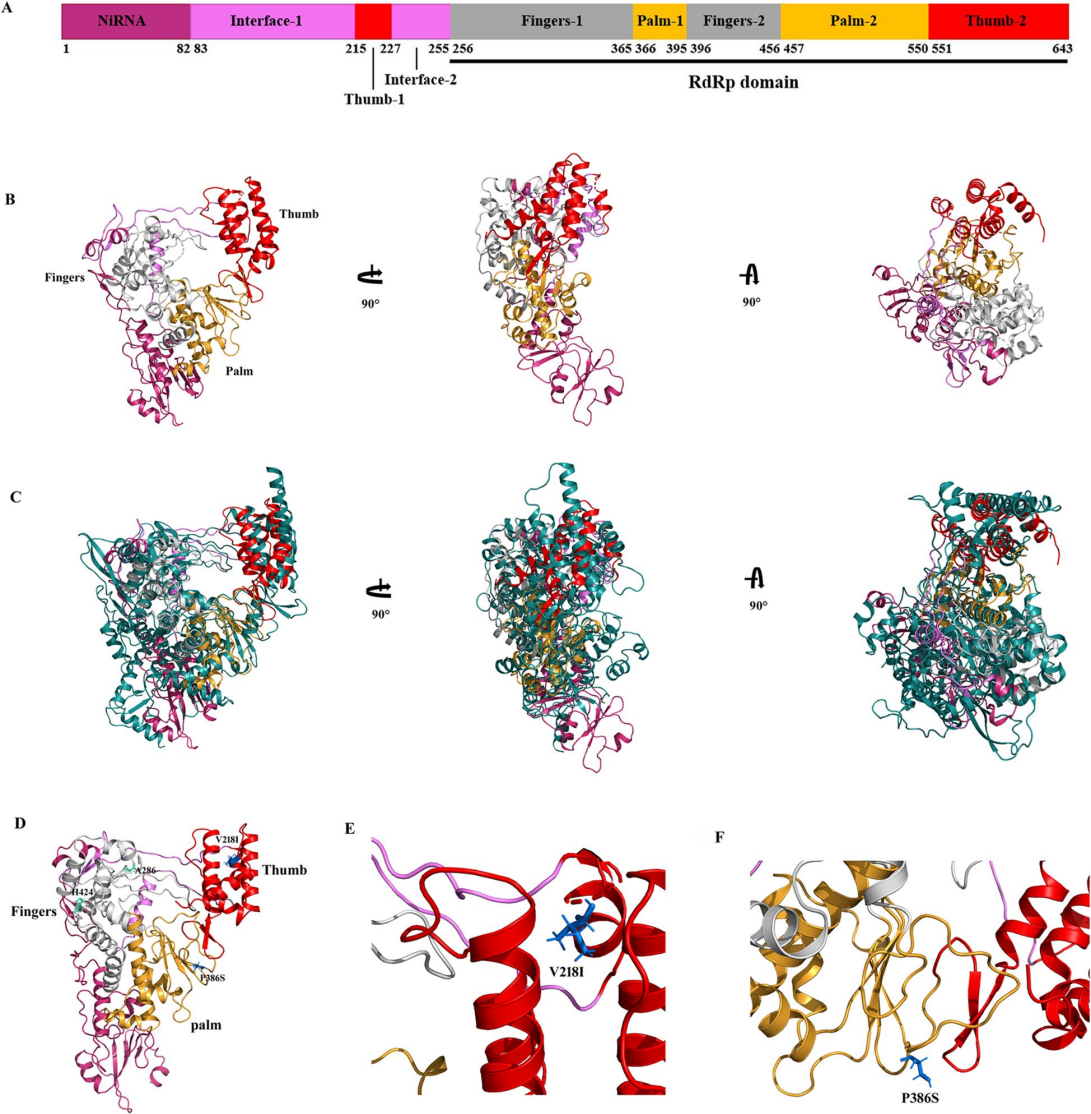
**Predicted model of NSP9 and structure-based modulation of viral RdRp fidelity and ribavirin resistance.** **A** Domain structure of NSP9 (RdRp). **B** Three views of the NSP9 structure generated by 90° rotations (left, back view; middle, side view; right, top view). **C** Aligned cartoon figure of PRRSV NSP9 and SARS-CoV-2 NSP12. The NSP12 is coloured in deep teal. **D** The positions of V218I and P386S (blue) and A286 and H424 (green-cyan), in the structural model. **E** Magnified cartoon figure of V218I. **F** Magnified cartoon figure of P386S. Colour code for NSP9 (NiRAN, interface, fingers, palm, and thumb) used throughout.

The online prediction tool AlphaFold Colab (Version 2.1.0) was used to simulate the three-dimensional (3D) structure of RdRp of NJ-Rc. The predicted structure was then compared with results from the DALI server. Our results found that the structure of PRRSV RdRp was highly similar to that of severe acute respiratory syndrome coronavirus 2 (SARS-CoV-2) RdRp (NSP12) (PDB ID: 6YYT) (Figure 8). In terms of the structural model of SARS-CoV-2 [27], we predicted that the RdRp (NSP9) of PRRSV would consist of at least two domains, an N-terminal domain with RdRp-associated nucleotidyltransferase (NiRAN) activity and a canonical RdRp occupying its C terminal domain. The PRRSV RdRp domain also included the fingers, palm, and thumb domains.

To address the above expectation, we inserted Thumb-1 into the interface domain, dividing it into interface-1 and interface-2 (Figure 8A). The 3D structure of PRRSV RdRp resembled a cupped right-hand structure [9], almost identical to the structure of SARS-CoV-2 NSP12 (Figures 8B and C). Consequently, the two critical sites for replication fidelity and ribavirin-resistance mutations found in this study were V218I in the thumb domain (Thumb-1), located in the centre of three α-helices, and P386S in the loop of the palm region (Figures 8D–F). Moreover, the ribavirin-resistance sites Ala283 and His421 reported by Tian et al. [8] correspond to the Ala286 and His424 in the RdRp of PRRSV NJ-Rc and are located in the palm region (Figure 8D).

## Discussion

Ribavirin, a guanosine nucleotide analog, is a mutagenic reagent that can be incorporated into RNA viral genomes during RNA synthesis because their RdRp lacks proofreading and repair mechanisms [22, 28]. Several RNA viruses, such as PRRSV [8, 18], have demonstrated resistance to ribavirin. This resistance has been attributed to the enhanced ability linked to the increased replication fidelity of their RdRp [11–13, 15]. In our study, two novel amino acid substitutions (V218I and P386S) were identified in the RdRp of PRRSV under ribavirin pressure. Ribavirin resistance has been associated with two amino acid substitutions, A283T and H421Y; conversely, in contrast to the ribavirin resistance sites reported by Tian et al. [8], we identified ribavirin resistance sites at Ala283 and His421 in PRRSV strain VR2385 and strain MN184B. Meanwhile, the corresponding RdRp in PRRSV NJ-Rb showed no mutations at Ala286 and His424 (Figure 7). However, Amina et al. selected two ribavirin-resistant PRRSV strains (RVRp13 and RVRp22, which were also the high-fidelity strains of VR23323 passaged under ribavirin pressure) and demonstrated no variation in the sequence alignment of RdRp [18].

As a result, whether these variations are linked to different strains remains ambiguous. However, this possibility is unlikely, given that the amino acids of RdRp are highly conserved across strains (Figure 7). If the variations in ribavirin-resistance sites are due to differences between different strains, further investigation is needed to determine whether specific viral structural or non-structural proteins influence the development of ribavirin resistance. Furthermore, amino acid substitutions in fidelity variants have been mapped to different positions of the viral RdRp, indicating that different residues are involved in determining RdRp fidelity [29].

Studies have reported that mutations in the 3D polymerase caused by ribavirin pressure result in the emergence of drug-resistant PV strains with high-fidelity replication and a low replication rate. These findings were later confirmed in enterovirus A71 (EV-A71) [8, 17, 30] and PRRSV [8, 17, 30]. In experiments to rescue ribavirin-resistant mutant viruses, the viral titres of PRRSV P30 remained ~ 2-log lower than that of the control virus without ribavirin. It is worth noting that these quasispecies variants contain potentially high-fidelity mutants. Moreover, the reconstituted viruses, vI218V, vS386P, and vVP, displayed lower viral peak titres than the parental virus vNJ-Rb (Figure 3B). In this study, the PRRSV NJ-Rb strain demonstrated higher replication fidelity compared to the wild-type. The ribavirin-resistant PRRSV may replicate at a slower rate, resulting in fewer lethal mutations over a given period, allowing it to avoid “error catastrophe”.

In addition, not all ribavirin-resistant mutants originated from high-fidelity strains (Figure 1). Ribavirin was used to select a ribavirin-resistant population of FMDV, and four amino acid substitutions (D5N, A38V, M194I, and M296V) were identified in the RdRp. However, the D5N, A38V, and DAMM (a mutant carrying all 4 amino acid substitutions) mutants were high-fidelity RdRp variants, but the M194I and M296V mutants were not [12]. While the clones selected in this study may not all contain V218I and P386S mutations, a guanidine assay was used to screen high-fidelity mutant strains, as we did not sequence each clone. Predictably, NJ-Rb produced lower progeny titres under guanidine pressure because fewer guanidine-resistance mutations were generated because of its higher replication fidelity than NJ-Rc.

To our knowledge, G64S PV is the first known example of a picornavirus with increased fidelity in RNA synthesis. Notably, the mutagenic activity of ribavirin (R) on PV is exerted after its intracellular conversion to the triphosphate form (ribavirin triphosphate, RTP), which the PV RdRp incorporates, and functions as a mutagenic purine analogue [28]. In this study, the mutant spectra of PRRSV populations passaged in the presence of ribavirin exhibited elevated mutation frequencies, indicating the continued mutagenic activity of ribavirin. Our findings show a highly significant increase in the proportion of the transitions C → U and G → A compared to other mutation types (data not shown). These findings suggest that PRRSV RdRp prefers incorporating RTP instead of GTP (guanosine triphosphate, GTP) rather than replacing ATP (adenosine triphosphate, ATP). Furthermore, UTP (uridine triphosphate, UTP) is incorporated more frequently than CTP (cytidine triphosphate, CTP) when R is present in the template RNA [31]. However, as the increased fidelity of the NJ-Rb virus was observed in the guanidine-resistance assay (Figure 2), it is unlikely that the sole determinant of ribavirin resistance is a specific reduction in RTP binding or incorporation. These above possibilities are not mutually exclusive and can be tested by determining the rates of RTP incorporation for NJ-Rb (V218I and P386S) and wild-type polymerases [23].

The ribavirin-resistant PRRSVs generated in this study possess restricted quasispecies diversity (Figure 2). For example, ribavirin-resistant viral populations display increased RdRp fidelity during RNA replication, resulting in reduced quasispecies sequence diversity. Mutation frequency can be high when a low-fidelity RdRp is present. In a complex environment, generating quasispecies may enable virus populations to respond and adapt rapidly [22]. Moreover, fidelity is typically adjusted to balance virus replication rates, pathogenesis, and tissue tropism necessary for virus growth [15]. However, we observed that the high-fidelity NJ-Rb did not replicate in PAMs, and its tissue tropism was significantly altered (Table 4 and Figure 4A). However, similar to the parental virus NJ-Rb, the P1 of recombinant viruses vI218V, vS386P, vVP, and vNJ-Rb did not induce a CPE in PAMs and were unable to replicate (Figure 4A).

However, we also found that the P30 of recombinant viruses could replicate in PAMs and produce a CPE (data not shown). Consequently, this finding indicated that the ribavirin-resistance sites of RdRp are not involved in regulating tissue preference. Furthermore, the viral quasispecies is not simply a collection of diverse mutants but a group of interactive variants that contribute to the population’s characteristics [7, 11]. Therefore, viral populations, rather than individuals, are the target of evolutionary selection. Notwithstanding, the proposal that high-fidelity strains could be vaccine candidates is not in doubt [7, 18, 22, 32]. The mutant (NJ-Rb) was almost incapable of establishing an infection or effectively replicating in the lungs (Figure 4C), even though the lungs are the primary site of wild-type PRRSV replication. For example, the immunisation of pigs with NJ-Rb has demonstrated robust protection against the PRRSV NJ-a strain (data not shown). Furthermore, residue Val218 was conserved among all selected PRRSV strains (Figure 7). Therefore, V218I in the high-fidelity PRRSV NJ-Rb strain could serve as a molecular marker to distinguish between natural infections. It is also important to note at this juncture that it is crucial to study the mutation suspected of affecting fidelity in a genetically clean background, meaning one with no other mutations present in the genome [21].

In our study, the results of the infectious cDNA clones demonstrated that both V218 and P386 were critical amino acid sites affecting the replication fidelity and ribavirin sensitivity of PRRSV, particularly V218 (Figures 3D, E, 6C). Furthermore, the sequence alignment data indicated that amino acid residues Val218 and Pro386 of RdRp are conserved in both PRRSV genotypes (Figure 7). The rescued reconstituted virus vS386P showed lower mutation frequencies than the other reconstituted virus vI218V (Figures 3D and E). Our study also found that residue Val218 exhibits higher conservation than residue Pro386. Consequently, it is reasonable to hypothesise that the V218I substitution is more important in conferring high fidelity. These findings were also confirmed by the infectious clones of PRRSV containing the mutations (V218I and P386S) constructed from the wild-type strain NJ-a P80 (Figure 6). However, Tian et al. [8] found that the ribavirin-resistance sites were Ala283 and His421, corresponding to Ala286 and His424, in PRRSV NJ-Rb RdRp. Whether Ala286 and His424 are also ribavirin-resistance sites of PRRSV NJ-Rb remains to be investigated by reverse genetics.

Over the past decade, there have been remarkable advances in our understanding of RdRps’ structure and function. These advances include discovering a mechanism for closing the active site that allows these viruses to easily fine-tune replication fidelity and quasispecies distribution [9, 15]. Thus far, the data support the belief that viral RdRps (positive-strand RNA virus polymerases) evolved and retained the unique palm domain-based movement for active-site closure [15]. However, based on the crystal structure of EV71 RdRp, L123 locates at the entrance of the RNA template-binding channel, which has been identified as a fidelity checkpoint [22]. In addition, the structurally equivalent RdRp in the PV G64S and FMDV G62S mutants has similar effects on the structure of the N-terminus pocket [9]. Moreover, there appear to be fidelity effects linking the N-terminus binding pocket to the roof of the nucleotide triphosphate entry tunnel through an unknown mechanism.

Interestingly, this study’s critical site for the PRRSV fidelity checkpoint was V218 in the thumb domain (Thumb-1), not the palm domain (Figure 8). The viral RdRps share a folding interaction where the fingertips (i.e., the top of the finger domain) reach across the active site to contact the top of the thumb. This finger-thumb contact is preserved across all positive-strand RNA virus RdRp structures and is important in maintaining structural integrity [9]. RdRp Leu420 in PV, situated on an α-helix of the thumb domain within the product RNA exit channel, is essential for RNA recombination and RNA recombination, which plays a significant role in ribavirin resistance [33]. Although elongation rates and the fidelity of RNA synthesis were largely unaffected by the alanine substitution mutations in the thumb α-helix, S416A exhibited slightly higher-fidelity RNA synthesis than other thumb α-helix mutants. This outcome suggests that the thumb domain is involved in regulating ribavirin sensitivity and replication fidelity.

Furthermore, PRRSV thumb-1 is inserted into the interface domain, dividing into interface-1 and interface-2 (Figure 8A). Briefly, the interface domain acts as a protein interaction junction and contacts the NiRAN domain, the fingers domain, and the other subunit [34]. The interface domain also contains a versatile and adaptable inter-domain linker that facilitates the adoption of multiple conformations during virus replication [35]. The replication fidelity and ribavirin resistance of PRRSV may be explained further by a hypothetical mechanism. For instance, V218 is located at the centre of the three α-helices in the thumb, similar to the active site in the palm. We predicted that the V218I substitution mutation stabilises PRRSV RdRp-RNA interactions within the polymerase elongation complex. Furthermore, we reasoned that RdRp-V218 could be involved in a previously unidentified fidelity checkpoint in PRRSV; however, the structural details require further investigation, particularly regarding the protein structure–function relationships.

P386 is located in the loop of the palm region of PRRSV RdRp rather than at the palm active site. The structural domains of RdRp, including the palm, finger, and thumb, are, in turn, composed of the classic motifs A–G arising from sequence conservation. The palm active site is built on a three-stranded β-sheet consisting of one strand from motif A and two from motif C, packed against a long α-helix from motif B [9]. Whether a different RNA proofreading mechanism is involved in the replication fidelity and ribavirin sensitivity of PRRSV replication fidelity and ribavirin remains unknown. In addition, the recombinant virus with the double mutation (I218V and S386P) did not show increased genetic instability (Figures 3 and 6), suggesting a possible lack of synergy between the two mutations. Previous studies have described high-replication-fidelity variants in PRRSV [8, 18] and other RNA viruses [11–13, 15]; however, these mutations are in different regions of the viral RdRp. Therefore, it is suggested that RNA viruses have different fidelity checkpoints consisting of multiple residues, which may work alone or cooperatively [22].

In summary, we identified two novel amino acid residues (Val218 and Pro386) in the RdRp of PRRSV. These residues are critical in determining PRRSV’s genetic stability and ribavirin resistance. This finding will help us better understand the molecular mechanisms underlying PRRSV evolution and pathogenesis, and it will aid in developing safer PRRSV vaccines.

## Data Availability

All the data generated during the current study are included in the manuscript.
